# Ultra-distal tibial fractures: a retrospective comparison of distal plate versus nail fixation

**DOI:** 10.1186/s10195-025-00832-3

**Published:** 2025-03-20

**Authors:** Feng Wang, Xiaoshu Zhu, Xiangyang Dai, Lei Wang, Chengpu Zhong, Jian Qin, Tangbo Yuan

**Affiliations:** 1 Department of Orthopedics, Sir Run Run Hospital, Nanjing Medical University, Nanjing, China; 2 Department of Medical Genetics, Nanjing Medical University, Nanjing, China

**Keywords:** Intramedullary nail (IMN), Distal tibial plate (DTP), Ultra-distal tibial fracture (UDTF), Retrospective study

## Abstract

**Background:**

Current literature on ultra-distal tibial fractures (UDTF) is relatively limited, particularly regarding the outcomes and complications of different treatment strategies, with data being notably scarce. This study aimed to compare the clinical outcomes of intramedullary nailing (IMN) and distal tibial plate (DTP) fixation in the treatment of UDTF.

**Methods:**

A total of 48 eligible patients were retrospectively reviewed and divided into two matched groups based on age, gender, injury severity score, and fracture type. The IMN group comprised 21 patients, and the DTP group included 27 patients. All patients were followed up to assess both clinical and radiological outcomes.

**Results:**

The IMN group demonstrated significantly shorter surgery time (*P* = 0.043) and fracture healing time (*P* = 0.002) compared with the DTP group. However, no significant differences were found between the two groups in terms of time from fracture to admission (*P* = 0.740), preoperative hospital stay (*P* = 0.310), postoperative hospital stay (*P* = 0.379), infection rates (*P* = 1.000), or rates of nonunion (*P* = 0.822). Postoperative malalignment occurred in three patients in the IMN group and one patient in the DTP group (*P* = 0.430). The mean postoperative angulation in both groups was similar in the coronal plane (*P* = 0.101) and sagittal plane (*P* = 0.334). The mean Olerud–Molander Ankle Score (OMAS) was 88.62 ± 5.24 in the IMN group and 85.85 ± 8.39 in the DTP group (*P* = 0.169).

**Conclusion:**

Both implants are effective in treating UDTF. However, IMN offers advantages in reducing surgical time, accelerating fracture healing, and promoting early recovery. Therefore, IMN may represent a superior surgical option for managing UDTF.

## Introduction

Tibial fractures are among the most prevalent lower limb injuries, with distal fractures commonly resulting from high-energy trauma, such as motor vehicle collisions or falls from height, as well as low-energy mechanisms such as simple falls [1, 2]. Ultra-distal tibial fractures (UDTF), a clinically significant subtype, are defined as extraarticular fractures occurring within 5 cm of the tibial articular surface without direct joint involvement. Although the term UDTF is not yet widely standardized in the orthopedic literature, it is used in this study to specifically describe fractures occurring at this unique anatomical location. This designation serves to distinguish UDTF from other distal tibial fractures, highlighting the distinct fracture line characteristics and its proximity to the ankle joint [3]. The ultra-distal tibia presents particular challenges owing to limited soft tissue coverage and relatively poor vascularization, complicating surgical management. Major complications associated with UDTF include infection, nonunion, and malunion [4].

Owing to the limitations of early intramedullary nails (IMN) in achieving stable fixation in the distal segment [5], many surgeons previously favored distal tibial plates (DTP) for treating these fractures. DTP, a traditional approach, has been widely used in clinical practice. It allows for direct visualization and precise fracture reduction, potentially reducing the risk of nonunion [6]. However, DTP often requires extensive dissection of the surrounding soft tissues and periosteum, which can compromise local blood supply, delay healing, increase blood loss, and elevate the risk of wound infection [7, 8]. With the advent of new-generation IMN, such as low-profile, multidirectional nails, treatment options for UDTF have expanded [9]. These nails can be implanted after closed reduction or through a minimally invasive incision, reducing soft tissue dissection and blood loss at the fracture site. This approach lowers the risk of soft tissue complications and infection while promoting faster fracture healing. However, the use of IMN may also increase the risk of malalignment [10, 11].

IMN and DTP are widely employed for the treatment of UDTF, yet the optimal approach remains a subject of ongoing debate [12]. Current literature on UDTF is relatively limited, particularly regarding the outcomes and complications of different treatment strategies, with data being notably scarce. Previous studies on distal tibial fractures have often overlooked the critical factor of the fracture line’s distance from the ankle joint surface. To more accurately assess the efficacy of IMN and DTP in this specific subset of fractures, this study focuses on patients with major fracture lines located 3–5 cm from the ankle joint. By comprehensively comparing the clinical and radiological outcomes of the two techniques, this study aims to provide a more precise evaluation of their respective roles in the management of UDTF.

## Materials and methods

This study was conducted at Sir Run Run Hospital, Nanjing Medical University. Ethical approval was obtained from both the Research Management Department and the Ethics Committee of Sir Run Run Hospital, Nanjing Medical University.

The inclusion criteria for this study were as follows: (1) ultra-distal tibial fractures, with the fracture line located 3–5 cm from the ankle joint; (2) closed fractures or Gustilo grade I or II open fractures; (3) age between 18 and 80 years; and (4) early failure of conservative treatment.

Using data from medical records and hospital databases (February 2019 to February 2023), we collected comprehensive information on age, sex, surgery type, and hospitalization details. Patients were matched based on sex, age, distance from the fracture line to the ankle joint, Injury Severity Score, and fracture pattern (AO classification). A total of 21 patients who underwent IMN were compared with 27 patients who received closed reduction and internal fixation with plating.

Surgical technique: All surgeries in this study were performed by a single senior orthopedic surgeon (T.B.Y.), serving as the lead surgeon, with over 10 years of specialized experience in ankle and lower limb surgeries. For all cases, a standardized supine position was employed to ensure consistency in patient positioning and adherence to uniform intraoperative protocols.

**Fig. 1:**
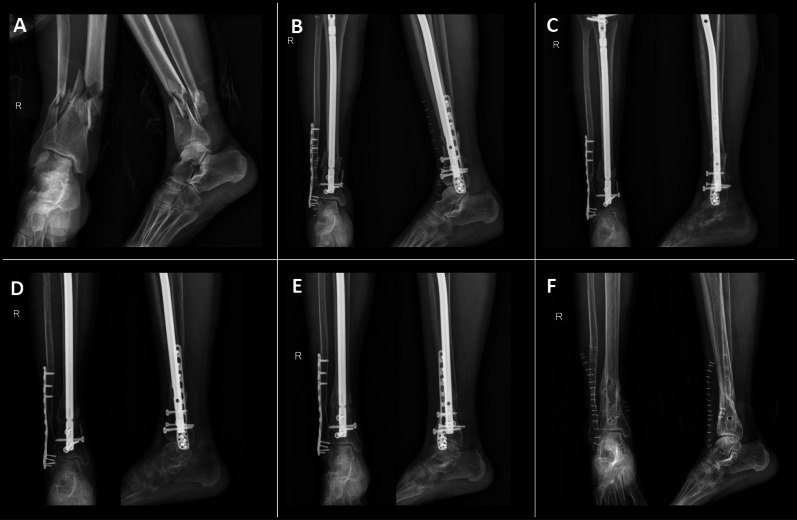
Patient with UDTF treated with IMN: **A** Right-sided ultra-distal tibia and fibula fractures; **B** Postoperative radiograph at 1 day; **C** Radiograph at 3 months postoperatively; **D** Radiograph at 6 months postoperatively; **E** Radiograph at 1 year postoperatively; **F** Radiograph following implant removal

IMN procedure: A longitudinal incision was made from the tibial tuberosity to the lower pole of the patella. Layers of tissue were dissected sequentially, reaching the superior aspect of the tibial tuberosity. Under fluoroscopic guidance, an entry point was created proximal to the tibial tuberosity, and a guidewire was introduced into the medullary canal, crossing the fracture site. The medullary canal was reamed using a flexible reamer, followed by the selection and insertion of an appropriately sized interlocking IMN under fluoroscopic control. To enhance distal stability, a blocking screw was inserted laterally at the distal end of the nail, and both proximal and distal interlocking screws were placed. If fibular fixation was required, the fracture site was exposed, reduced, and fixed with a locking plate. The surgical site was irrigated with diluted povidone–iodine solution and normal saline, followed by meticulous hemostasis and layered closure. The procedure was completed successfully, and the patient was transferred to the recovery ward postoperatively (Expert Tibial Nail, Zhengtian, Tianjin, China) (Fig. 1).

**Fig. 2:**
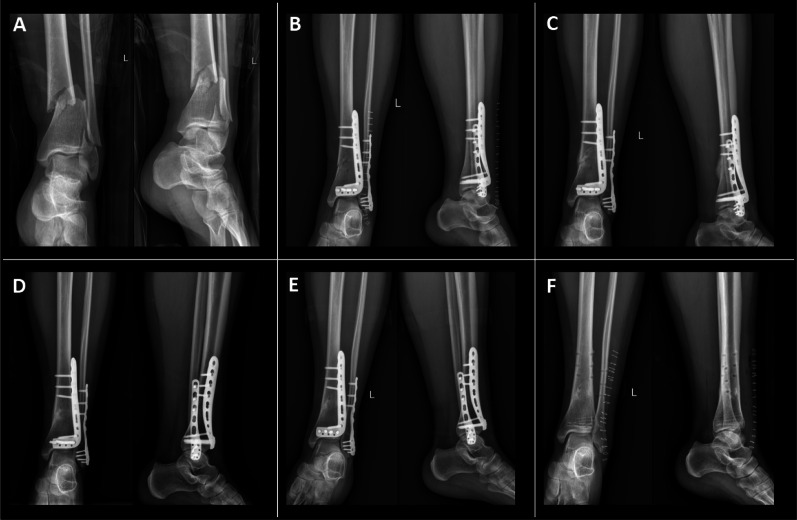
Patient with UDTF treated with DTP: **A** Left-sided ultra-distal tibia and fibula fractures; **B** Postoperative radiograph at 1 day; **C** Radiograph at 3 months postoperatively; **D** Radiograph at 6 months postoperatively; **E** Radiograph at 1 year postoperatively; **F** Radiograph following implant removal

Plating procedure: A longitudinal incision was made at the midpoint between the tibial crest and the anterior border of the fibula. Layers of tissue were dissected sequentially, and the incision was extended along the medial edge of the tibialis anterior tendon to expose the UDTF. Hematoma and interposed soft tissue at the fracture site were debrided, and fracture reduction was achieved. An appropriately sized DTP was selected and placed on the anterolateral aspect of the tibia. Under fluoroscopic guidance, holes were drilled sequentially, and screws were inserted for fixation. If fibular fixation was required, the fracture site was exposed, reduced, and fixed with a locking plate. The surgical site was irrigated with diluted povidone–iodine solution and normal saline, followed by meticulous hemostasis and layered closure. The procedure was completed successfully, and the patient was transferred to the recovery ward postoperatively (Distal Tibia Anterolateral locking Plate, Zhengtian, Tianjin, China) (Fig. 2).

Patients in both groups were administered a third-generation cephalosporin, such as cefazolin, as a prophylactic antibiotic within 30–60 min prior to surgery. Postoperatively, cefazolin was continued via intravenous infusion for an additional 2 days, depending on patient condition and assessed infection risk. On postoperative day 1, patients were encouraged to engage in ankle joint mobility exercises and isometric quadriceps strengthening exercises. On postoperative day 2, gradual initiation of active and assisted knee flexion exercises was commenced. Clinical and radiographic follow-ups were conducted every 6 weeks until fracture healing was confirmed. The criteria for fracture healing included the absence of pain or tenderness at the fracture site, the patient’s ability to bear full weight without pain, and the presence of bridging callus on radiographs taken from at least two different angles. Healing occurring within 6–9 months was classified as delayed union, whereas fractures that did not heal within 9 months were classified as nonunions [13]. Radiographic assessments, including anteroposterior and lateral X-rays, were performed to evaluate angular deformities at the fracture site. Malunion was defined as an angular deformity exceeding 5° in any plane [14]. Functional recovery was assessed using the Olerud–Molander Ankle Score (OMAS): a score above 91 was categorized as excellent, 61–90 as good, 31–60 as fair, and below 30 as poor [15].

### Statistical analysis

All statistical analyses were performed by an independent statistician using SPSS version 25.0. The Kolmogorov–Smirnov and Shapiro–Wilk tests were used to assess the normality of the data. Homogeneity of variances was evaluated with Levene’s test. For continuous variables following a normal distribution, results are expressed as mean and standard deviation. For non-normally distributed continuous variables, the Mann–Whitney *U* test was applied, and data are reported as median and interquartile range (IQR). Categorical variables were compared between groups using the chi-squared test or Fisher’s exact test, as appropriate.

## Results

The study enrolled 48 patients, consisting of 32 men and 16 women, with a mean age of 50.3 years (range, 20–68 years). Patients were distributed between the IMN and DTP groups, with no statistically significant differences in baseline characteristics, including age, gender distribution, or distance from the fracture line to the ankle joint (*P* > 0.05). Fractures were categorized according to the AO/OTA classification system. A detailed summary of the demographic characteristics for both groups is provided in Table 1.

**Table 1:** Demographic and operative data of the two groups

		Fixation type, no. of patients (%)		
		IMN group (*n* = 21)	DTP group (*n* = 27)	*P* value
Sex	Male	14 (66.7)	18 (66.7)	1.000
	Female	7 (33.3)	9 (33.3)	
Age, mean (SD), years		50.0 (12.1)	50.6 (13.2)	0.693
Fracture line to ankle joint distance, mean (SD), m		3.9 (0.7)	3.6 (0.6)	0.168
Associated fibula fracture	Yes	21 (100)	26 (96.3)	1.000
	No	0 (0)	1 (3.7)	
Fixation of fibula	Yes	8 (38.1)	17 (63.0)	0.087
	No	13 (61.9)	10 (37.0)	
Open fractures (Gustilo type 1 or 2)		2 (9.5)	0 (0)	0.186
Classification (AO/OTA)	A1	7 (33.3)	8 (29.6)	0.962
	A2	8 (38.1)	11 (40.7)	
	A3	6 (28.6)	8 (29.6)	

In this study, 47 patients (97.9%) presented with concomitant fibular fractures (Table 1). Among these, 8 patients in the IMN group and 17 patients in the DTP group underwent fibular fixation. Statistical analysis revealed no significant difference between the groups regarding the impact of fibular fixation on the healing of UDTF (*P* = 0.087). Additionally, two patients in the IMN group sustained Gustilo–Anderson type I or II open fractures. Given the relatively low severity of these injuries and the risk of pin tract infection associated with temporary external fixation, calcaneal traction was chosen as an interim stabilization method, followed by definitive IMN fixation after 5 and 7 days, respectively. Infection control and wound evaluation were performed prior to definitive surgery to ensure suitability for fixation. The incidence of open fractures did not differ significantly between the IMN and DTP groups (*P* = 0.186).

Compared with the DTP group, the IMN group exhibited a statistically significant reduction in both surgical duration and fracture healing time (*P* < 0.05) (Table 2). No significant differences were observed between the groups regarding pre-admission fracture time, preoperative hospital stay, or postoperative hospital stay (*P* > 0.05). Notably, the IMN group had no reported cases of postoperative infection, whereas one patient in the DTP group developed a site-specific infection necessitating implant removal. Despite this occurrence, the difference in infection rates between the groups was not statistically significant (*P* > 0.05). The infected patient received appropriate intervention, including removal of the internal fixation device, temporary external fixation, and antibiotic therapy. Subsequent to infection resolution, a secondary procedure was performed to insert a new DTP. All patients successfully recovered after the second surgery, with no further complications. In the IMN group, two patients experienced nonunion, which was effectively managed with additional plating and bone grafting on the IMN, resulting in successful fracture healing. Conversely, one patient in the DTP group also encountered nonunion, but achieved healing following bone graft treatment. Regarding deformities, 21.1% of patients in the IMN group had malalignment exceeding 5°, with one case of varus deformity and two cases of procurvatum. In comparison, the DTP group had 16% of patients with malalignment exceeding 5°, with no cases of varus deformity and one case of procurvatum. The mean postoperative angulation in the coronal plane (varus/valgus) was 2.50° (IQR: 2.09–2.93°) for the IMN group and 1.98° (IQR: 1.67–2.76°) for the DTP group, with no significant intergroup difference (*P* = 0.101). In the sagittal plane, the mean postoperative procurvatum/recurvatum was 2.09° (IQR: 1.45–2.81°) in the IMN group and 1.64° (IQR: 1.33–2.16°) in the DTP group, with similarly no significant difference (*P* = 0.334). The average OMAS was 88.62 ± 5.24 for the IMN group and 85.85 ± 8.39 for the DTP group, with no statistically significant difference observed between groups (*P* = 0.169).

**Table 2:** Comparative results of clinical and radiological outcome

		IMN group (*n* = 21)	DTP group (*n* = 27)	*P* value
Pre-admission fracture time, median (IQR),days		1 (0–2)	1 (0–3)	0.740
Preoperative stay, mean (SD), days		7.30 (3.16)	6.43 (2.54)	0.310
Operating time, mean (SD), min		102.05 (17.19)	110.70 (11.54)	0.043
Postoperative stay, median (IQR), days		3 (3–4)	3 (2–3.5)	0.379
Union time, mean (SD), weeks		18.29 (3.48)	21.59 (3.78)	0.002
Infection		0	1	1.000
Delayed union/nonunion		2	1	0.822
Malalignment (> 5°)		3	1	0.430
Malalignment	Coronal plane, median (IQR)	2.50 (2.09–2.93)	1.98 (1.67–2.76)	0.101
	Sagittal plane, median (IQR)	2.09 (1.45–2.81)	1.64 (1.33–2.16)	0.334
OMAS	Average score, mean (SD)	88.62 (5.24)	85.85 (8.39)	0.169
	Excellent	10	12	0.827
	Good	11	15	0.827
	Fair	0	0	–
	Poor	0	0	–

*IQR* interquartile range, *OMAS* Olerud–Molander Ankle Score

## Discussion

The management of UDTF presents significant challenges owing to the limited soft tissue coverage, compromised blood supply, and proximity to the ankle joint [1]. Current surgical options primarily include IMN, plating, and external fixation [4, 16]. Once soft tissue conditions are stabilized, IMN and DTP are commonly employed as definitive treatment strategies for these fractures [1].

In this study, we controlled for the distance between the fracture line and the ankle joint surface, a parameter that has seldom been statistically compared in prior research on these two surgical approaches. This factor is clinically significant, as it influences both the complexity of surgery and subsequent treatment outcomes [17]. Fractures closer to the ankle joint are particularly challenging owing to limited bone stock, altered load distribution, and thinner soft tissue coverage, which increase the risks of infection, nonunion, and implant failure [18]. In such cases, DTP may provide better alignment and facilitate early rehabilitation but carries a higher risk of wound complications due to greater soft tissue exposure. By contrast, IMN minimizes soft tissue disruption and preserves periosteal blood supply, offering advantages when sufficient bone stock is available for distal locking screws [19]. Biomechanical studies suggest that IMN with two distal locking screws provides adequate fixation strength for fractures near the joint, with a reduced infection risk [17]. In our study, the mean fracture line distance was 3.6 cm in the DTP group and 3.9 cm in the IMN group. The slightly shorter distance in the DTP group was not statistically significant (*P* = 0.168), thus ensuring the comparability of the two techniques. These findings underscore the critical role of fracture line distance in optimizing surgical decisions and outcomes. Notably, nearly all patients with UDTF in our study also presented with concomitant fibular fractures. The role of fibular fixation in UDTF remains a topic of ongoing debate. While fibular fixation may not significantly influence functional outcomes in UDTF, it could potentially improve fracture reduction stability, albeit at the cost of an increased risk of nonunion [20–22].

In this study, the majority of patients with UDTF were admitted to our hospital within 24 h of injury, with a few patients receiving initial care at local hospitals before being transferred for further treatment. There were no significant differences in the time from fracture to admission and preoperative hospitalization duration between the IMN and DTP groups (*P* > 0.05), suggesting similar fracture severity and preoperative swelling periods between the groups. This comparability enhances the robustness of our analysis of the two surgical approaches. Although previous studies have reported significant differences in postoperative hospital stays, favoring IMN for reducing socioeconomic burden and healthcare costs [9], our findings did not demonstrate a shorter postoperative stay for the IMN group compared with the DTP group (*P* = 0.379). This discrepancy may be attributed to factors such as postoperative management protocols, bed turnover rates, and patient preferences. Therefore, we propose that postoperative hospitalization duration may not serve as a primary comparative metric between these two surgical approaches in some hospital settings.

Research has indicated that IMN may facilitate accelerated fracture healing and earlier resumption of daily activities due to reduced soft tissue damage [23]. Our study corroborates this finding, with the IMN group demonstrating a significantly shorter fracture healing time compared with the DTP group, at 18.29 weeks versus 21.59 weeks (*P* = 0.002). Deformity healing is commonly defined as an angular deviation exceeding 5° in the coronal or sagittal plane, or 15° in the horizontal plane [14]. Numerous studies have highlighted the advantages of DTP in providing superior reduction and stability for UDTF, which may promote bone healing [3, 24, 25]. In contrast, IMN permits controlled micromovements at the fracture site, which stimulate callus formation and secondary bone healing through endochondral ossification, particularly in diaphyseal fractures [26]. However, in ultra-distal fractures, excessive micromotion may lead to instability and misalignment, underscoring the critical importance of accurate reduction and fixation [27]. This dynamic reflects the complex biomechanical trade-off between promoting healing tissue formation and maintaining optimal fracture stability. IMN also faces challenges in achieving precise anatomical reduction, especially near the diaphyseal-metaphyseal junction, owing to the mismatch between the implant and the tibial medullary canal. Additionally, inadequate locking screw grip may result in device malpositioning, compromising alignment and increasing the risk of deformity healing [26, 28]. Reported malalignment rates for IMN range from 5% to 20%, depending on the fracture type and the surgical technique used. These technical challenges can lead to suboptimal outcomes, including malunion and delayed union, which have been associated with long-term functional limitations in some studies [29, 30].

Recent advancements in IMN design and auxiliary techniques have addressed some of these issues. For example, multidirectional distal angle-stable screws and blocking screws have proven effective in maintaining reduction and alignment. Blocking screws applied in the anterior–posterior direction can mitigate coronal plane deformity, while those applied in the medial–lateral direction can alleviate sagittal plane deformity [31]. In our study, one patient in the IMN group experienced varus deformity, and two patients experienced anterior apex deformity, resulting in deformity healing (> 5°). In contrast, one patient in the plating group had anterior apex deformity, also leading to deformity healing (> 5°). There were no statistically significant differences between the two surgical methods in terms of deformity healing (*P* = 0.430). Furthermore, the average postoperative angulation was comparable between the two groups, with no significant differences in the coronal (*P* = 0.101) and sagittal (*P* = 0.334) planes. These findings suggest that, compared with traditional DTP, the new IMN provides similar alignment maintenance for UDTF, with most angular deviations remaining within acceptable limits.

Delayed and nonunion of UDTF pose significant clinical challenges, with inadequate blood supply to the surrounding soft tissues being a key factor contributing to delayed healing or nonunion. IMN offers notable advantages in treating UDTF by preserving the integrity of surrounding soft tissues and vascular supply, thus promoting biological healing [26]. However, the larger medullary canal in the distal tibial metaphysis presents technical difficulties in achieving stable fixation, which may partially explain the marginally lower union rate observed with IMN. In addition to these technical challenges, factors such as patient bone quality (e.g., osteoporosis), variations in postoperative immobilization protocols, and individual compliance may further influence union rates [28, 32]. Our study found a 90.5% union rate in the IMN group and a 96.3% union rate in the plating group, with no statistically significant difference (*P* = 0.822). This suggests that, despite technical difficulties, IMN yields healing outcomes comparable to DTP, with no significant differences in delayed healing or nonunion rates. However, the observed difference in union rates warrants further investigation into the underlying contributing factors. Notably, numerous studies have shown that IMN and plating techniques result in similar fracture healing rates and complication incidences, which aligns with the findings of our study [25].

The infection rate following UDTF surgery varies widely, ranging from 0% to 50% [14, 33]. Some studies suggest that DTP may carry a higher risk of wound infection compared with IMN. This increased risk is likely due to the larger soft tissue incision required for plate placement, which may cause periosteal stripping and disrupt the local blood supply. Additionally, prolonged mechanical irritation from the plates may also contribute to the higher infection risk [34, 35]. In our study, the infection rate was 0% in the IMN group and 3.7% in the DTP group, with no statistically significant difference (*P* > 0.05), consistent with previous findings [9]. Both groups in our study strictly adhered to infection prevention protocols, including preoperative antibiotic administration, meticulous soft tissue handling, and postoperative wound care. These measures likely contributed to the overall low infection rates. Although infections in the DTP group were successfully managed without long-term consequences, these findings underscore the importance of infection prevention and minimizing soft tissue trauma during surgery.

Regarding functional outcomes, prior research has indicated no significant difference in OMAS scores between the IMN and DTP groups at 6 months postoperatively [36]. Our study also utilized the OMAS scoring system to evaluate functional recovery. The results demonstrated no statistically significant differences in functional outcomes between the two surgical methods (*P* = 0.169). This suggests that both IMN and DTP achieve comparable clinical results, with patient functional recovery falling within acceptable ranges regardless of the chosen surgical approach.

## Conclusions

This study concludes that there were no significant differences between IMN and DTP in terms of fracture time, hospital stay, infection rates, ankle range of motion, or rates of malunion and nonunion. Both implants effectively treated UDTF, providing stable rigid fixation to prevent secondary fracture collapse. However, IMN demonstrated superior performance in reducing operative time, accelerating fracture healing, and facilitating early return to daily activities. Thus, IMN may be considered a preferable surgical option for managing UDTF.

## Data Availability

All the data in the study are available from the corresponding author on reasonable request.

## Abbreviations

IMN: Intramedullary nail
DTP: Distal tibial plate
UDTF: Ultra-distal tibial fracture
IQR: Interquartile range
OMAS: Olerud–Molander Ankle Score
